# Preventive effects of sodium hyaluronate combined with pelvic floor neuromuscular electrical stimulation on the intrauterine adhesions in women after abortion

**DOI:** 10.17305/bb.2023.9467

**Published:** 2024-02-01

**Authors:** Yanan Sun, Wenjuan Zhang, Yuru Cai, Haiyan Li

**Affiliations:** 1 Department of Gynecology and Obstetrics, Bethune International Peace Hospital, Shijiazhuang, China; 2 Pelvic Floor Rehabilitation Clinic, Shijiazhuang People’s Hospital, Shijiazhuang, China; 3 Department of Gynecology, Shijiazhuang People’s Hospital, Shijiazhuang, China

**Keywords:** Intrauterine adhesions (IUAs), sodium hyaluronate, pelvic floor neuromuscular electrical stimulation (NMES), abortion, surgery

## Abstract

The aim of this study was to investigate the clinical efficacy of combining pelvic floor neuromuscular electrical stimulation treatment (NMES) with sodium hyaluronate in preventing intrauterine adhesions (IUAs) following abortion. A total of 140 women who underwent artificial abortion were enrolled. The control group received only an intrauterine injection of sodium hyaluronate post-surgery, while the observation group received both the injection and daily pelvic floor NMES treatments, beginning on the day after the abortion. Monthly follow-ups on menstrual conditions were conducted for six months post-surgery. Fasting venous blood samples from both groups were collected on the second day post-abortion and the day after treatment. Transvaginal color Doppler ultrasound was used on the second day post-abortion and the 15th day post the first menstrual cycle to measure endometrial thickness, and the pulsatility and resistance indices of the endometrial spiral arteries. Over the six-month follow-up, the combination therapy group exhibited a notably lower IUA incidence compared to the control group (2.8% vs 15.7%). Furthermore, combined treatment significantly expedited post-abortion menstrual recovery, reduced vaginal bleeding volume and duration (*P* < 0.001). It also increased endometrial thickness and reduced the endometrial spiral artery’s pulsatility and resistance indices (*P* < 0.05). In addition, lower serum tumor necrosis factor alpha (TNF-α) and higher interleukin-10 (IL-10) were found in the observation group compared to the control group (*P* < 0.05). The combination therapy offers significant advantages in preventing and reducing IUA after abortion, resulting in a substantial reduction in IUA occurrence.

## Introduction

Intrauterine adhesion (IUA), commonly referred to as Asherman’s syndrome, is a functional disorder of the uterine cavity that poses significant challenges for women of reproductive age. IUA results from damage to the endometrial basal layer, preventing its regeneration and potentially leading to partial or complete occlusion of the cervical canal or/and uterine cavity [1]. This condition manifests as scar tissue formation within the uterine cavity following various uterine traumas, such as surgical interventions or infections [2]. Women with IUA often face risks of abnormal menstrual cycles, amenorrhea, infertility, and recurrent miscarriages, with consequent detrimental impacts on their health [2–4].

Recently, there has been growing concern regarding the increased prevalence of IUA in women post-abortion. Abortion procedures may elevate the risk of IUA due to the associated trauma [5, 6]. Moreover, post-abortion infections can intensify the likelihood of IUA by inducing inflammation and subsequent scarring. Given the recent introduction of the third-child policy in China and advancements in diagnostic technology, it is anticipated that IUA detection and diagnosis rates will continue to rise. As such, understanding IUA’s etiology, ensuring accurate diagnoses, offering effective treatments, and preventing adhesion recurrence are paramount.

The pursuit of effective IUA treatments remains a pivotal topic in gynecology. Hysteroscopic IUA separation has emerged as a prevalent treatment modality for IUA, with the potential to reinstate the normal anatomical structure and function of the endometrium [7]. However, it is crucial to acknowledge that the surgical procedure might inadvertently damage adjacent tissues, possibly precipitating adhesion recurrence post-surgery. Hyaluronic acid sodium gel, a variant of hyaluronic acid, has gained traction in clinical settings for endometrial repair [8]. Its ability to stabilize and insulate the uterine cavity cultivates an optimal environment for tissue regeneration. Furthermore, pelvic floor neuromuscular electrical stimulation (NMES) is leveraged to fortify pelvic floor muscles. This electrical stimulation invigorates the pelvic floor muscles, augmenting blood flow to the region, thereby facilitating healing and curtailing scar tissue formation [9]. This research aims to delve into the clinical efficacy of combining pelvic floor NMES with sodium hyaluronate to prevent IUA post-abortion.

## Materials and methods

### Inclusion and exclusion criteria

Inclusion criteria include patients aged 18–35 who had regular menstrual cycles ranging from 26 to 30 days, amenorrhea lasting between 42 and 60 days, and a confirmed intrauterine pregnancy as evidenced by B-ultrasound on the day of the therapeutic abortion. As per our records, we did not encounter any embryos lacking a fetal heartbeat.

Exclusion criteria include patients with IUA, genetic and chromosomal abnormalities, tumors, severe actual genital diseases, liver and kidney dysfunction, poor compliance or communication barriers, mental health disorders, congenital reproductive organ developmental abnormalities, hematological disorders, contraindications or allergies to pelvic floor electromyography treatment, local skin lesions or ulcers, and chronic illnesses.

### Participants

Between December 2020 and December 2022, we enrolled a total of 140 patients who chose to terminate their pregnancies and underwent artificial abortion in our hospital. The patients were randomly divided into a control group and an observation group using a random number table, with 70 patients in each group. Both groups experienced painless abortions and received routine post-operative antibiotics to prevent infections. Advisories against sexual intercourse, swimming, and bathing were in place for one month post-surgery, and any deviations or abnormalities were promptly addressed. Post-operation, the control group was administered a single 5 mL injection of sodium hyaluronate. In contrast, the observation group received both the sodium hyaluronate injection and pelvic floor NMES treatment.

### Pelvic floor neuromuscular electrical stimulation treatment

The pelvic floor NMES treatment began on the first day after the artificial abortion. On the first post-operative day, electrode pads were placed at the midpoint between the navel and xiphoid process and the corresponding point on the back, above the pubic symphysis and at the level of the third sacral vertebra on both sides of the groin. A vaginal probe was inserted when there was no bleeding from the vagina. The frequency selection spanned three stages.

Initially, a modulated current with a frequency of 1/4/1 Hz and a pulse width of 270/230/270 µs was deployed with no delays, rise, drop, or rest times, and a 30-s plateau. Subsequently, a modulated current at a frequency of 40/80/40 Hz and a pulse width of 320/280/320 µs was employed, featuring no delay, a 3-s rise time, a 3-s plateau, a 2-s drop time, and a 9-s rest interval. In the final stage, a modulated current at 3 Hz frequency with a 150 µs pulse width was used. This stage had no delay, rise, drop, or rest times but included a 5-s plateau. The electrical stimulation’s intensity was adjusted to ensure the patient’s comfort and should not cause pain, typically ranging between 10 and 40 mA. The treatment was administered daily, each session lasting about 30 min, over a consistent period of 15 days.

### Measurement and follow-ups

The demographic characteristics of the participants are detailed in Table 1. Post-surgical abortion, the onset of the first menstrual period, including the volume and duration of bleeding, was documented. Menstrual conditions (such as menstrual volume, cycle length, and duration) were monitored in monthly follow-ups for six months post-surgery. In instances of irregularities, like reduced menstrual flow, endometrial thickness under 6 mm, or amenorrhea, a hysteroscopy was conducted to evaluate the potential occurrence of IUA.

**Table 1:** Demographic and clinical characteristics of women received the treatments of sodium hyaluronate (control) or sodium hyaluronate combined with pelvic floor neuromuscular stimulation (observation) after abortion

**Characteristics**	**Control (*n* ═ 70)**	**Observation (*n* ═ 70)**	***P* value**
Age (years)	26.86 ± 4.21	27.19 ± 4.47	0.293
BMI (kg/m^2^)	22.05 ± 3.37	22.72 ± 3.84	0.131
Gestational weeks	7.82 ± 1.13	7.56 ± 1.48	0.397
*Gravidity, (%)*			
0	38 (54.3)	35 (50.0)	0.589
1	16 (22.9)	13 (18.6)	
2	12 (17.1)	14 (20.0)	
>2	4 (5.7)	8 (11.4)	
*Abortion history, (%)*			
0	48 (68.6)	45 (64.3)	0.741
1	12 (17.1)	16 (22.9)	
2	9 (12.9)	7 (10.0)	
>2	1 (1.4)	2 (2.8)	
*Uterine curettage history, (%)*			
Yes	15 (21.4)	19 (27.1)	0.555
No	55 (78.6)	51 (72.9)	

The data presented are mean ± SD or *n* (percentage). The comparisons of data between the two groups were done by Mann–Whitney test or chi-square test or Fisher’s exact test. BMI: Body mass index.

On the day following the induced abortion and two days post-treatment, fasting venous blood samples (3 mL) were drawn from both patient groups. Initially, the blood was collected in an EDTA-coated tube, after which serum was separated via centrifugation. The serum concentrations of tumor necrosis factor alpha (TNF-α) and interleukin-10 (IL-10) were quantified using enzyme-linked immunosorbent assays with kits sourced from Abcam.

On the second day post-induced abortion and 15 days following the first menstrual cycle post-abortion, a transvaginal color Doppler ultrasound was employed. This measured the thickness of the endometrium and evaluated the pulsatility index and resistance index of the spiral arteries embedded within the endometrium.

### Diagnosis criteria of intrauterine adhesion

IUA was classified into mild, moderate, and severe adhesion according to adhesion coverage. Mild adhesion presents as thin filmy adhesion, covering less than 1/4 of the uterine cavity. It rarely involved the upper part of the cervical canal or the tubal ostia, ensuring the tubal ostia remain unaltered. Moderate adhesion involves adhesions on the uterine cavity wall spanning an area between 1/4 and 3/4. This might partially occlude the upper section of the uterine cavity and some of the tubal ostia. Severe adhesion indicates an increase in the thickness of the adhesion on the uterine cavity wall, occupying more than 3/4 of the area. This results in the total occlusion of the upper section of the uterine cavity and all of the tubal ostia.

### Ethical statement

The study was approved by the Ethics Committee of Shijiazhuang People’s Hospital (#LCYJ-V603-2). The study was performed in strict accordance with the Declaration of Helsinki, Ethical Principles for Medical Research Involving Human Subjects. Written consent was obtained from the participant’s parents or guardians.

### Statistical analysis

Statistical Package for the Social Sciences (SPSS) software version 22.0 was used for data sorting and analysis. The comparisons of data between the two groups were made by Fisher’s exact test. Quantitative variables were shown in mean and standard deviation, and qualitative variables were expressed in terms of number and ratio. The categorical variables were performed by unpaired *t*-test with Welch’s correction and Mann–Whitney test. Two-way ANOVA followed Tukey’s multiple comparisons test was performed to compare various groups. *P* value less than 0.05 was considered statistically significant.

## Results

### Basic characteristics

The demographic and clinical characteristics of the 70 patients in the control group and the 20 patients in the observation group are presented in Table 1. Following the procedure, the control group was administered a 5 mL intrauterine injection of sodium hyaluronate, whereas the observation group received the pelvic floor NMES treatment in addition to this injection. Data regarding gravidity, previous abortions, and history of uterine curettage were collected prior to the current pregnancy and abortion. The average age for the control group was 26.86 ± 4.21 years, and for the observation group, it was 27.19 ± 4.47 years. No significant differences were observed between the two groups in terms of age, BMI, gestational weeks, gravidity, history of abortion, and uterine curettage (*P <* 0.05).

### Sodium hyaluronate combined with pelvic floor NMES treatment effectively reduced the frequency of IUA after abortion

Menstrual conditions for both the control and observation groups were monitored monthly for six months post-surgery. This monitoring included evaluations of menstrual volume, period, and duration (Table 2). Over the 6-month follow-up period, 11 cases of IUA were reported in the control group, which consisted of 6 mild cases, 3 moderate cases, and 2 severe cases. In contrast, the observation group reported only two cases of IUA, with one mild and one moderate case. The total incidence of IUA was significantly different between the two groups: 15.7% in the control group and 2.8% in the observation group. Notably, the incidence of IUA in the observation group was significantly less than that in the control group (*P* ═ 0.017).

**Table 2:** Comparison of intrauterine adhesions occurrence between women received the treatments of sodium hyaluronate (control) or sodium hyaluronate combined with pelvic floor neuromuscular stimulation (observation) after abortion

**Groups**	**Mild**	**Moderate**	**Severe**	**Total occurrence**
Control (*n* ═ 70, %)	6 (8.6)	3 (4.3)	2 (2.8)	11 (15.7)
Observation (*n* ═ 70, %)	1 (1.4)	1 (1.4)	0 (0)	2 (2.8)
*P* value	–			0.017

The data presented are *n* (percentage). The comparisons of data between the two groups were done by Fisher’s exact test.

**Figure 1.**
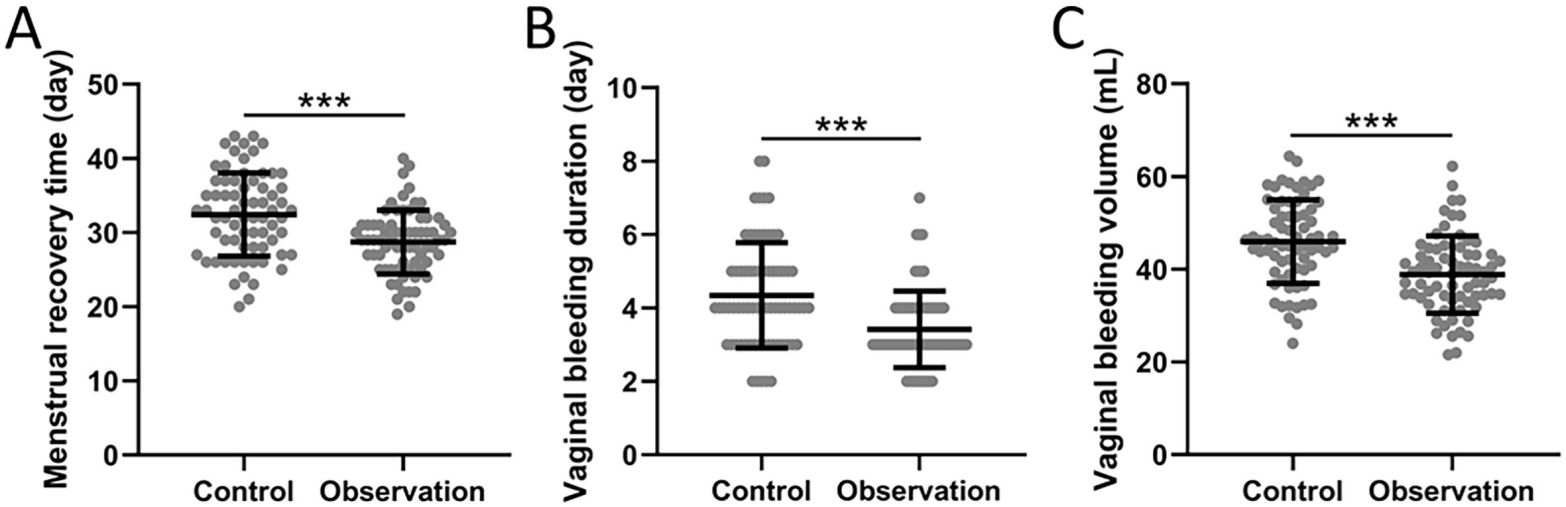
**Comparisons of menstrual recovery time (A), vaginal bleeding duration (B), and vaginal bleeding volume (C) between women received the treatments of sodium hyaluronate gel (control) or sodium hyaluronate combined with pelvic floor neuromuscular stimulation (observation) after abortion.** Data are shown with mean ± SD. ***P < 0.001 from Mann–Whitney test or unpaired *t*-test with Welch’s correction.

**Figure 2.**
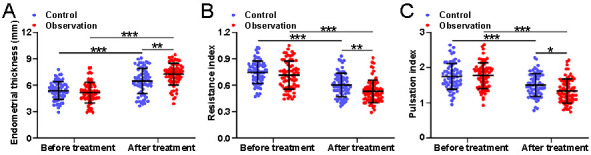
**Comparisons of endometrial thickness (A), resistance index (B), and pulsation index (C) before and after the treatments between women received the treatments of sodium hyaluronate gel (control) or sodium hyaluronate combined with pelvic floor neuromuscular stimulation (observation) after abortion.** Data are shown with mean ± SD. *P < 0.05, **P < 0.01, ***P < 0.001 from two-way ANOVA followed by Tukey’s multiple comparisons test.

### Sodium hyaluronate combined with pelvic floor NMES treatment effectively improved menstrual and endometrial recovery

Comparing the time, amount, and duration of the first menstruation after the current abortion between the two groups, we observed that the combined treatment of sodium hyaluronate and pelvic floor NMES significantly shortened the time of post-abortion menstrual recovery (Figure 1A), decreased the duration of vaginal bleeding (Figure 1B), and reduced the amount of vaginal bleeding (Figure 1C) compared to the control group (all *P* < 0.001).

Furthermore, we analyzed the measurements of endometrial thickness (Figure 2A), endometrial spiral artery RI (Figure 2B), and PI (Figure 2C) using transvaginal color Doppler ultrasound on the second day post-abortion and on day 15 of the first post-operative menstrual period, comparing the observation group to the control group. Our findings revealed that the combination of sodium hyaluronate and pelvic floor NMES treatment was effective in increasing the endometrial thickness and decreasing the endometrial spiral artery PI and RI compared to the control group (*P <* 0.05).

### Sodium hyaluronate combined with pelvic floor NMES treatment effectively reduced the concentration of inflammatory factors

**Figure 3.**
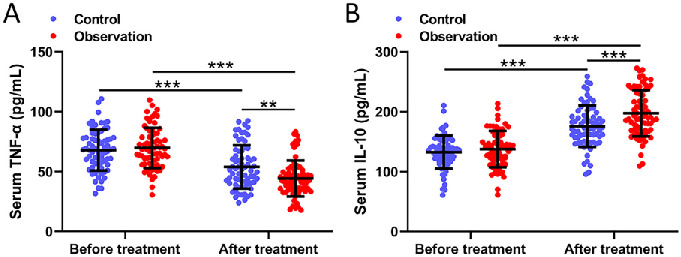
**Comparisons of serum TNF-α (A) and IL-10 (B) before and after the treatments between women received the treatments of sodium hyaluronate gel (control) or sodium hyaluronate combined with pelvic floor neuromuscular stimulation (observation) after abortion.** Data are shown with mean ± SD. **P < 0.01, ***P < 0.001 from two-way ANOVA followed by Tukey’s multiple comparisons test. TNF-α: Tumor necrosis factor alpha; IL-10: Interleukin-10.

We measured the concentrations of TNF-α and IL-10 in patients’ serum on the second day post-surgery and the second day following the end of treatment across both groups. Comparisons of serum TNF-α (Figure 3A) and IL-10 (Figure 3B) concentrations before and after the treatments between the two groups were illustrated. Our results underscored that the concentration of serum TNF-α in the observation group was significantly lower than that in the control group. Conversely, the serum IL-10 concentration in the observation group saw a notable rise when juxtaposed against the control group (*P* < 0.05). Inflammation has been closely associated with the onset and progression of IUA. The synergistic treatment approach of sodium hyaluronate and pelvic floor NMES proved more effective than that of the control group in diminishing the concentration of inflammatory markers and augmenting the body’s anti-inflammatory response.

## Discussion

IUA predominantly arises from endometrial damage due to surgery and/or post-abortion infection, which can lead to irregular menstrual cycles. Clinically, patients often report symptoms, such as abdominal pain and inconsistent menstruation. In the absence of timely intervention, complications like amenorrhea and infertility can develop [10].

Pelvic floor NMES is a non-invasive treatment that employs electrical currents to activate the pelvic floor muscles, enhancing their strength and coordination [11, 12]. It is a prevalent remedy for several pelvic floor disorders, including urinary incontinence, fecal incontinence, and pelvic pain [13]. IUA manifests when scar tissue develops within the uterus, often stemming from trauma or injury to the uterine lining [14]. Such scars can cause the uterine walls to adhere, leading to issues ranging from menstrual irregularities to infertility and recurrent miscarriages. Pelvic floor NMES, by stimulating pelvic muscles and enhancing regional blood circulation, aids in fostering healing and curbing scar tissue development [9, 15].

Sodium hyaluronate is a natural component of the extracellular matrix and is found in high concentrations in connective tissues, including the uterus [16]. Hyaluronan (HA) plays a significant role in regenerative medicine, particularly in preventing IUAs after procedures like artificial abortion and laparoscopic endometriosis surgery, as it promotes tissue healing and regeneration [17–20]. Additionally, in gynecology, HA is commonly used as a biocompatible material for medical devices, such as intrauterine device with HA coatings, to reduce inflammation and enhance biocompatibility [21]. Moreover, HA is used in lubricants and vaginal moisturizers to alleviate vaginal dryness, particularly during menopause or postpartum periods [22]. Studies have shown that it has anti-inflammatory and anti-adhesive properties, making it an effective treatment for IUA. In addition, it can effectively stabilize and isolate the uterine cavity and associate with endometrium repairment. Therefore, we proposed a study to explore the effectiveness of combining pelvic floor NMES with sodium hyaluronate to prevent IUA in women post-abortion.

Within our study, the control group was administered an intrauterine sodium hyaluronate injection post-surgery. In contrast, the observation group was treated with pelvic floor NMES alongside the injection. Over the 6-month follow-up, those subjected to the combined therapy exhibited a notably reduced IUA incidence compared to their sodium hyaluronate-only counterparts. Concurrently, data revealed that the amalgamated sodium hyaluronate and pelvic floor NMES treatment could effectively expedite post-abortion menstrual recovery, minimize vaginal bleeding volume, and curtail bleeding duration relative to the control group. The combined regimen also demonstrated efficacy in augmenting endometrial thickness and reducing endometrial spiral artery PI and RI values. TNF-α, an inflammatory cytokine, and IL-10, its anti-inflammatory counterpart, were observed [23, 24]. The observation group manifested lower serum TNF-α and elevated IL-10 levels compared to controls, suggesting that the combination treatment curtailed inflammatory markers while bolstering the body’s anti-inflammatory defense. Hence, the synergy of sodium hyaluronate and pelvic floor NMES proves promising in preventing IUA development post-abortion.

The treatment of sodium hyaluronate combined with pelvic floor neuromuscular stimulation presents an attractive option for women seeking to minimize the risk of IUAs following an abortion. This therapy is relatively simple and can be performed in an outpatient setting, making it accessible to a wide range of women.

Despite the promising results, there are limitations to consider. The follow-up time of six months may not capture long-term effects. Additionally, the small sample size and single-center setting could impact the generalizability of the findings. Addressing these issues in future research would enhance the reliability and applicability of the combination therapy’s observed effects.

## Conclusion

IUA can be a debilitating condition resulting from trauma during an abortion. The treatment of sodium hyaluronate combined with pelvic floor NMES showed significant benefits in reducing IUA post-surgery, improving post-operative recovery, and potentially impacting women’s reproductive health by increasing endometrial thickness and regulating inflammatory responses. This study could provide valuable insights into effective prevention and treatment strategies for IUA. However, further research is needed to fully understand the mechanisms of action of this therapy and to optimize its use in clinical practice.
